# GITR expression on *T*-cell receptor-stimulated human CD8^+^ T cell in a JNK-dependent pathway

**DOI:** 10.4103/0971-6866.60188

**Published:** 2009

**Authors:** Subhasis Chattopadhyay, Nitya G. Chakraborty

**Affiliations:** 1 Department of Medicine, University of Connecticut Health Center, Farmington, CT 06030 - 1628, USA; 2 School of Biological Sciences, National Institute of Science Education and Research, Institute of Physics Campus, PO: Sainik School, Bhubaneswar, Orissa - 751 005, India

**Keywords:** GITR, human CD8^+^ T cell, JNK, *T*-cell receptor

## Abstract

Glucocorticoid-induced tumor necrosis factor receptor (TNFR) (GITR) family-related gene is a member of the TNFR super family. GITR works as one of the immunoregulatory molecule on CD4^+^ regulatory T cells and has an important role on cell survival or cell death in CD4^+^ T cells. Little is known about the expression of GITR on human CD8^+^ T cells on antigen-specific and non-specific activation. Here, we report that expression of GITR on human CD8^+^ T cells on *T*-cell receptor (TCR) (anti-CD3)-mediated stimulation is dependent on the JNK pathway. The activation of CD8^+^ T cells was measured by the expression of IL-2 receptor-α (CD25), GITR and by IFN-γ production upon re-stimulation with anti-CD3 antibody. We studied the signaling pathway of such inducible expression of GITR on CD8^+^ T cells. We found that a known JNK-specific inhibitor, SP600125, significantly down-regulates GITR expression on anti-CD3 antibody-mediated activated CD8^+^ T cells by limiting JNK phosphorylation. Subsequently, after stimulation of the CD8^+^ cells, we tested for the production of IFN-γ by the activated cells following restimulation with the same stimulus. It appears that the expression of GITR on activated human CD8^+^ T cells might also be regulated through the JNK pathway when the activation is through TCR stimulation. Therefore, GITR serves as an activation marker on activated CD8^+^ cells and interference with JNK phosphorylation, partially or completely, by varying the doses of SP600125 might have implications in CD8^+^ cytotoxic T cell response in translational research.

## Introduction

Glucocorticoid-induced tumor necrosis factor (TNFR) (GITR) family-related gene, a member of the TNFR super family (TNFRSF), was originally cloned in a glucocorticoid-treated hybridoma T cell line.[1-3] Many reports are available about its expression on CD4^+^ T cells as an important component of the immunoregulatory unit.[3,4] It has a high homology with other TNFRSF members.[1-5] CD4^+^ CD25^+^ regulatory T cells (T-reg) are known to express higher levels of surface GITR, which may regulate the immunosuppressive function of CD4^+^ T-reg.[2,3] It is also reported that GITR regulate activation induced cell death (AICD) in murine T cells and exert costimulatory function.[6] Most of the work on GITR has been performed on CD4^+^ cells in the murine system. Little is known about the expression of GITR on human CD8^+^ T cells. Analysis for the relative expression of GITR on CD8^+^ T cells may add new information in relation to cytotoxic T cell response with specific antigen in translational research, like inflammatory diseases and tumor immunity. Further, the functional aspects of CD8^+^ cells, e.g. IFN-γ secretion, cytolytic ability and the ability for self-destruction-AICD in relation to the signaling mechanisms involving GITR expression, if any, could add a very important point in immunotherapeutic intervention. Here, we observed the activation-induced expression of GITR on CD8^+^ T cells upon T-cell receptor (TCR) (anti CD3-Ab)-driven stimulation followed by functions. We found that GITR expression on activated CD8^+^ T cells is JNK (c-Jun NH(2)-terminal kinase) dependent, as evident by the down-regulation of GITR on activated CD8^+^ T cells when treated with the JNK-specific inhibitor (SP600125). The functional properties of activated CD8^+^ T cells with or without SP600125 were also tested by IFN-γ production upon restimulation. Thus, it appears that GITR expression on activated human CD8^+^ T cells and its signal may be regulated through the JNK pathway, indicating an importance of using a specific inhibitor in immunotherapy protocols.

## Materials and Methods

### Reagents

Monoclonal antibodies: Anti-human GITR FITC mAb, anti-human CD8 PE mAb, purified phopspho-specific JNK mAb, goat anti-mouse APC mAb and purified anti-human CD3mAb were purchased from BD Biosciences (San Jose, CA, USA). Inhibitors for various kinase pathways, such as SB203580 (SB) for p38 kinase, SP600125 (SP) for JNK and PD98059 (PD) for ERK were purchased from BIOMOL (Plymouth Meeting, PA, USA). Twenty-five micromolar concentration of the inhibitors, dissolved in DMSO, for various kinases, were used as described earlier.[7]

Peripheral blood lymphocytes (PBL): Human PBL were isolated on a Ficoll-Hypaque gradient as described earlier.[8,9] The participants were included in this study with informed consent.

### Cells and culture media

Tissue culture technique and the procedure have been described earlier.[8-10] Briefly, tissue cultures were performed in Iscove's medium (Life Technologies Inc., Grand Island, NY, USA) supplemented with 10% fetal bovine serum (Gemini Bio-Products Inc., Calabasas, CA, USA), L-arginine (0.55 mM), L-asparagine (0.24 mM) and L-glutamine (1.5 nM), (Life Technologies Inc.) henceforth described as complete medium (CM).

### Purification of CD8^+^ T cells

Human CD8^+^ T cells from PBL were isolated as described previously[8,9] as per the manufacturer's (Dynal, Oslo, Norway) protocol. Briefly, CD8^+^ T cells were positively purified from PBL using CD8-positive selection magnetic beads and detached using detached beads solution. The purity of CD8^+^ T cells was 94-98%, confirmed by FACS analysis. Phenotypic analysis

The immunofluorescence procedure for phenotypic analysis by flow cytometry was performed after 72 h of TCR stimulation (5 µg/ml soluble anti-human CD3 mAb and 2 mg/ml anti-human CD28 mAb treatment), mentioned in short as anti-CD3 antibody or TCR stimulation in the text. The method for phenotypic analyses in flow cytometry has been described previously.[8,9] Briefly, cell surface staining was carried out with fluorochrome-conjugated monoclonal antibodies for CD8, CD25 and GITR. Intracellular staining on CD8^+^ T cells for JNK was performed using phopspho-specific JNK mAb, appropriate secondary conjugated antibody and Cytofix/Cytoperm reagent (BD Biosciences).

### IFN-γ enzyme-linked immunosorbent assay

Supernatant from CD8^+^ T -cell culture (72 h) was kept in (–)70°C until use. The supernatants were assayed for IFN-γ in ELISA as per the manufacturer's (DuoSet ELISA Development System, RandD System) protocol, which is described earlier.[8,9]

## Results

**Figure 1:**
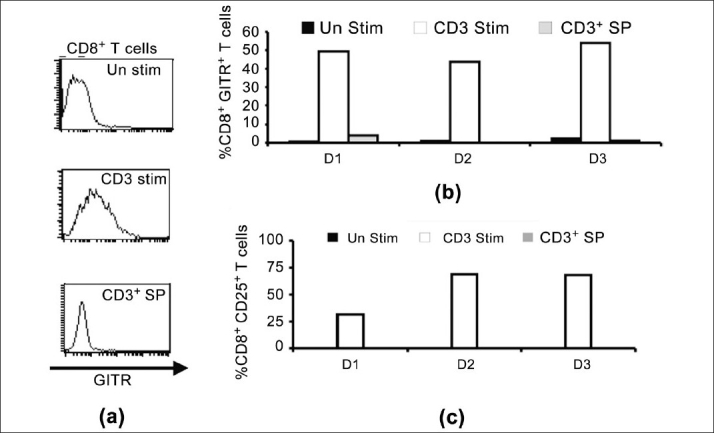
Regulation of human CD81 *T*-cell activation by the JNK inhibitor (SP) after *T*-cell receptor-driven stimulation. (a) Representative FACS data of glucocorticoid-induced tumor necrosis factor receptor (GITR) expression (mean fluorescence intensity) on human CD8^+^ T cells; (b) Graphical representation of FACS data of GITR expression (%) on human CD8^+^ T cells in three different cases; (c) Graphical representation of FACS data of CD25 expression (%) on human CD8^+^ T cells in three different cases (D1, D2, D3: Three different donors)

**Figure 2:**
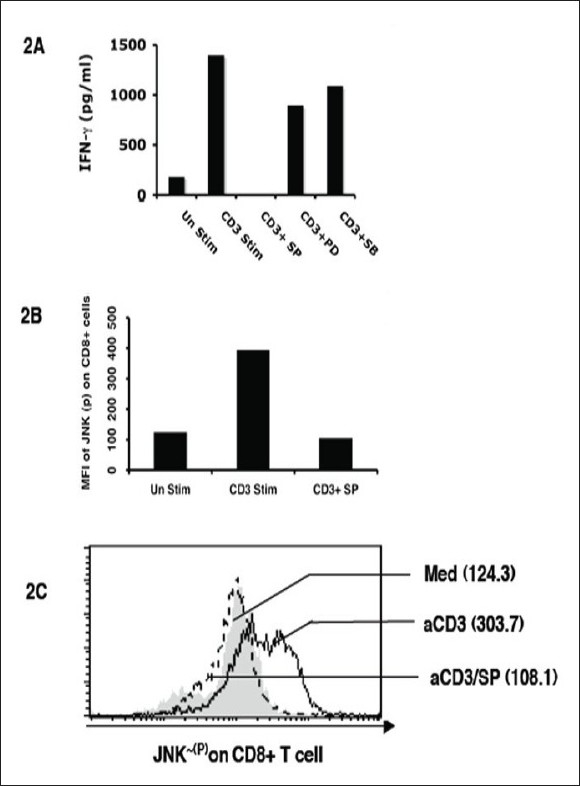
Regulation of human CD8^+^ *T*-cell function by JNK inhibitor after *T*-cell receptor-driven stimulation. (a) IFN-³ production (pg/ml) from human CD8^+^ T cells; (b) Graphical representation of FACS data (mean fluorescence intensity) of JNK-phosphorylation [JNK ~ [P]] on human CD8^+^ T cell; (c) FACS data of JNK-phosphorylation [JNK ~ [P]] on human CD8^+^ T cell. [SB203580 (SB) for p38 kinase, SP600125 (SP) for JNK and PD98059 (PD) for ERK inhibitors]

First, we studied the expression of GITR on human CD8^+^ T cells after stimulation with anti-CD3 antibody. We performed flow cytometric analysis of GITR on CD8^+^ T cells. After 72 h of initiation of cultures, significantly high levels of CD25 and GITR expression by CD8^+^ T cells [Figure 1] was evident. We also found similar results showing that anti-CD3 stimulation up regulates CD25 on a majority of the T cell subset (data not shown). As JNK is known to regulate IL-2Rα (CD25) on activated T cells,[11-13] we were interested to see whether the expression of GITR on activated CD8^+^ T cells involves any known signaling mechanism leading to the JNK pathway. Here, we used JNK-specific inhibitor SP600125 in combination with anti-CD3-driven stimulation. Interestingly, we observed that this JNK-specific inhibitor can down-regulate the GITR level significantly (> 70%) on activated CD8^+^ T cells [Figure 1]. Similar results were also obtained in CD4^+^ T cells (data not shown). The functional properties of activated CD8^+^ T cells on anti-CD3-driven stimulation (72 h) and its regulation by SP600125 were further studied by IFN-γ production and the corresponding JNK phosphorylation (JNK-^[P]^). Here, we found that both IFN-γ production and JNK phosphorylation (JNK-^[P]^) were significantly down-regulated by SP600125 [Figure 2].

## Discussion

The c-Jun amino terminal kinase (JNK) signaling pathways have been associated with cell death, differentiation and proliferation. After stimulation with antigen, CD4^+^ and CD8^+^ T cells show differential effector functions and thus control specific aspects of the immune responses. The studies carried out by different groups indicate that the role of JNK in CD4^+^ T cells is different from their role in CD8^+^ T cells. Moreover, these two pathways are not redundant in either T cell population. It has been shown that p38 MAP kinase regulates early stages of *T*-cell development in the thymus. It is therefore important to consider the specific function of these kinases in each T cell population when pharmacological inhibitors of JNK and p38 MAP kinases are used for therapeutic purposes to control the immune responses.

Recently, in the mouse model, it has been shown that GITR can costimulate along with TCR-driven stimulation in CD4^+^ CD25 T cells for ERK activation.[6] But, the involvement of JNK in human CD8^+^ T cells in relation to GITR expression is yet to be established. Here, we found the regulatory effect of JNK inhibitor (SP600125)[12] to down-regulate GITR on human CD8^+^ T cells [Figure 1]. During an immune response, the CD8^+^ T cells take a leading role in infection immunity and exert several effector functions[12-16] and also become the regulatory target in tumor immunity and autoimmunity.[8,15] It is possible that GITR, as one of the newly identified TNFR members, may have some critical immunoregulatory role on CD8^+^ T cells, which is highly important for further evaluation. There may be different signaling mechanisms, which may regulate GITR function during the activation process (specific or non-specific). Here, we report that activated human CD8^+^ T cells express GITR and that its expression may be regulated by the JNK pathway, which may have some further implication in translational research, like tumor immunity and inflammatory diseases, where CD8^+^ T cells are one of the key effector cells.
